# In search for non precious metal oxide electrodes with the case of BaMoO_3_ thin films for hydrogen evolution reaction

**DOI:** 10.1038/s41598-025-21707-x

**Published:** 2025-10-29

**Authors:** Phu Tran Phong Le, Vadim Ratovskii, Anja Bieberle-Hütter, Gertjan Koster, Christoph Baeumer

**Affiliations:** 1 MESA+ Institute for Nanotechnology, University of Twente, PO Box 217, Enschede, 7522 NH The Netherlands; 2 Dutch Institute for Fundamental Energy Research (DIFFER), P.O. Box 6336, Eindhoven, 5600 HH The Netherlands; 3 Peter Gruenberg Institute 7, Forschungszentrum Juelich GmbH, 52425 Juelich, Germany; 4 JARA-FIT, RWTH Aachen University, 52056 Aachen, Germany

**Keywords:** Catalysts, Electrolysis, Perovskite oxides, Hydrogen evolution reaction, Degradation, Thin films, Electrocatalysis, Electrocatalysis, Renewable energy

## Abstract

Non-precious metal oxides are inexpensive catalysts for hydrogen evolution reaction (HER) in water electrolysis to realize the global-scale green hydrogen production. Here, we report on perovskite BaMoO_3_ thin films fabricated by pulsed laser deposition. The metallic films, grown on single crystalline NdGaO_3_ substrates, have the expected stoichiometry, are crystalline, and epitaxially grown in (001) direction. Relatively low electrochemical activity is found and the thin films rapidly degrade under HER conditions, contrary to expectations from the literature. Our research highlights that the processing conditions and the processing related properties of a material can have considerable effect not only on the activity, but also on the stability. In particular, stability assessment is key in identifying promising, alternative HER catalysts. In search for alternative materials, degradation needs to be investigated from the very first and specifically include catalyst characterization after electrochemical aging experiments. Attention must be paid to investigate the intrinsic properties of a material, to be aware of processing related properties of a catalyst, and to complement the performance analysis with detailed materials characterization.

**Supplementary Information:**

The online version contains supplementary material available at 10.1038/s41598-025-21707-x.

## Introduction

Water electrolysis has been considered as one of the most promising technologies for large-scale clean hydrogen production using renewable electricity^1,2^. However, materials scarcity and costs have hampered large scale implementation^3^. In particular, the highest catalytic activity for the hydrogen evolution reaction (HER) is achieved with precious metal Pt-based catalysts, which are electrochemically highly active and long-term stable, but scarce and expensive. Therefore, less active Ni-based cathodes are usually employed in commercial alkaline electrolyzers and alternative materials have to be investigated for further optimization. Non-precious metal oxides might offer an alternative that is flexible in terms of composition, structure, and synthesis method. However, their catalytic activity for HER is still inferior compared to precious metal catalysts though advanced designs of complex shapes^4^, with well-structured porosity^5^, and heterostructures^6^ have recently been boosting HER activity and competitiveness. Many potentially highly-active non-precious metal oxide HER catalysts have been reported recently^7–14^.

It is known that metal oxides are in general very stable in alkaline environment. However, fundamental electrocatalyst research frequently does not address stability under reaction conditions specifically. Therefore, long-term stability of alternative catalysts often remains largely elusive which hampers commercial interests. In addition, most non-precious metal oxide HER catalysts have been studied mainly using wet chemical synthesis where the catalyst is provided as micro- and nano-sized powder and used in combination with binders and supporting materials, such as Nafion and carbon black. Such approach, appearing in first instance economically beneficial regarding the scalable synthesis methods, impedes identification of intrinsic catalyst activity and stability. Studies in other catalytic systems, like oxygen reduction and evolution reactions, have shown that the intrinsic catalytic properties can be enhanced from the interactions between catalysts and supporting materials, leading to the improvement of catalytic activity and stability^15^. Such effects have not been addressed sufficiently in HER catalyst systems. A point of attention is degradation due to mass loss of powder-based electrodes, which might remain undetected in short term lab experiments. However, such electrodes would likewise fail in commercial application.

To investigate intrinsic materials properties as well as stability, thin films fabricated by vacuum thin film deposition methods offer a possible route to identify stable catalysts with appropriate, intrinsic properties. Thin film systems have drawn attention to the investigation of intrinsic catalytic properties of catalysts in oxygen evolution reaction (OER) which is the other half reaction of water electrolysis next to HER^16^. Catalyst materials can, for instance, been grown as epitaxial single crystalline thin films on single crystalline substrates with specific orientation. This allows to evaluate the intrinsic OER activity and stability solely as a function of orientation which is not possible with multi-facet powders bound together with diverse chemical additions. In addition, the actual surface area of smooth thin films is well known and can be approximated with the geometric area^16,17^, while the actual surface area of powder-based electrodes is challenging to assess^18^. Thin film systems have also facilitated the improvement of structure-property-activity descriptors of OER electrocatalysts, deriving new design principles for the development of highly efficient OER powder-based catalysts^16,17^. In this sense, thin film systems are an ideal method to assess and to select non-precious metal oxide HER catalysts.

In this study, we examine the *4d* transition metal oxide perovskite BaMoO_3_ because it has been reported as a highly-active and stable HER catalyst^10^. While the material used by other researchers was processed by co-precipitation, reduction process, and casting of powder in the presence of binders on glassy carbon^10^, we employ pulsed laser deposition (PLD) to grow BaMoO_3_ thin films on single crystalline NdGaO_3_(001)_*pc*_ substrates (*pc* stands for pseudo-cubic). The aim of our study is to confirm the high activity and stability of BaMoO_3_, but in the form of thin films as starting point for more detailed research on alternative materials for HER. However, the results show that BaMoO_3_ (001) is extremely unstable which questions also the long-term stability of multi-facet BaMoO_3_.

Although a universal procedure of stability testing has not been established for thin film systems so far, our work suggests that the general phase and facet instability of non-precious metal oxide HER catalysts can be quickly assessed after CV cycles with thin films.

## Experiments

### Pulsed laser deposition of BaMoO_3_

Pulsed laser deposition (PLD) was performed in a system from TSST (Enschede, the Netherlands) with a base pressure of 2 × 10^− 8^ mbar equipped with a (Krypton Fluoride) KrF excimer laser of 248 nm, pulse width of 25 ns, modulation frequency of 50 Hz (COMPexPro from Coherent Inc., USA) and *in-situ* reflection high-energy electron diffraction (RHEED) (STAIB Instruments, Germany). The energy distribution of the laser is gaussian-like. A stoichiometric BaMoO_4_ target (1 inch/~25 mm in diameter, 99.99% purity, SurfaceNet, Germany) was used in combination with a growth temperature of 700 °C, substrate-target distance of 50 mm, rectangular (3:10) spot size of 1.8 mm^2^, fluence of 1.3 J/cm^2^, and laser-frequency of 2 Hz. The depositions were carried out in vacuum with a base pressure of 2 × 10^− 6^ mbar at 700 °C without background gas. Single crystalline NdGaO_3_(001)_*pc*_ (Shinkosha, Japan) substrates of 10 × 10 × 0.5 mm^3^ were used as received. The same growth conditions were used for the deposition of BaMoO_3_ films on native SiO_2_/Si substrates, which were covered on the surface by Ca_2_Nb_3_O_10_ nanosheets (more details in Supporting Information).

### Thin film characterization

Thin film X-ray diffraction (XRD) data were measured in Bragg-Brentano θ-2θ geometry using PANalytical X’Pert Pro MRD (Malvern Panalytical, Netherlands) with the selection of Cu Kα_1_ radiation from a commercial incoming beam Ge220 monochromator. BaMoO_3_ thin films were investigated by Atomic Force Microscope (AFM) (Bruker Dimension ICON, USA) operating in tapping mode with TESPA-V2 tip (Bruker) and high-resolution scanning electron microscopy (SEM) with In-lens secondary electron detector on a Merlin field emission microscope (Zeiss 1550, Germany) at electron high tension of 1.4 kV. The transport measurements were performed in the 4-probe van der Pauw configuration using the Quantum Design Physical Properties Measurement System “Dynacool” (Quantum Design, Germany).

### Electrochemical measurements

Electrochemical measurements were carried out in a rotating disk electrode set-up (RDE, Pine Research, USA). A custom-made adapter to hold the sample back side to the Pt plug of the rotating disk electrode was used^19^. Platinum (Pt) of 50 nm thickness was sputtered for ohmic contacts on the back side and the front side edges of the samples, employing a shadow mask. A circular film area of 0.289 cm^2^ (or 6.07 mm in diameter) was exposed to the electrolyte and sealed using an O-ring. The schematic of sample configuration for HER is described in Figure S1 in the Supporting Information (SI). The RDE shaft was rotating at 1600 rpm. Electrochemical testing was performed in an alkaline-resistant Teflon cell (Pine Research, USA) with a graphite counter electrode (Pine Research, USA) using a BioLogic VSP-300 potentiostat (BioLogic, France). The electrolyte solution of 0.1 M KOH was prepared by dissolving KOH pellets (Sigma-Aldrich, 99.99%) in deionized water (Milli-Q, > 18.2 MΩ cm). The electrolyte was bubbled with Argon for at least 30 min before testing and also during testing. All electrochemical measurements were performed at room temperature. Potentials were referenced to a Hg/HgO reference electrode (Pine Research, USA) and converted to RHE scale using the measured Hg/HgO potential of 0.889 V vs. RHE. All electrochemical testing was performed with a fresh electrode that had not undergone previous testing. Cyclic voltammetry (CV) was performed in the range of 0.4 V (the open circuit potential) to − 0.5 V vs. RHE at a scan rate of 10 mVs^− 1^. Current densities are referred to geometric areas of the BaMoO_3_ thin film. Inductively coupled plasma mass spectrometry (Agilent Technologies 7900 ICP-MS, USA) in the He collision cell mode was used to determine the amount of Ba and Mo in the electrolytes before and after HER.

## Results and discussion

### Growth of BaMoO_3_ thin films

**Fig. 1:**
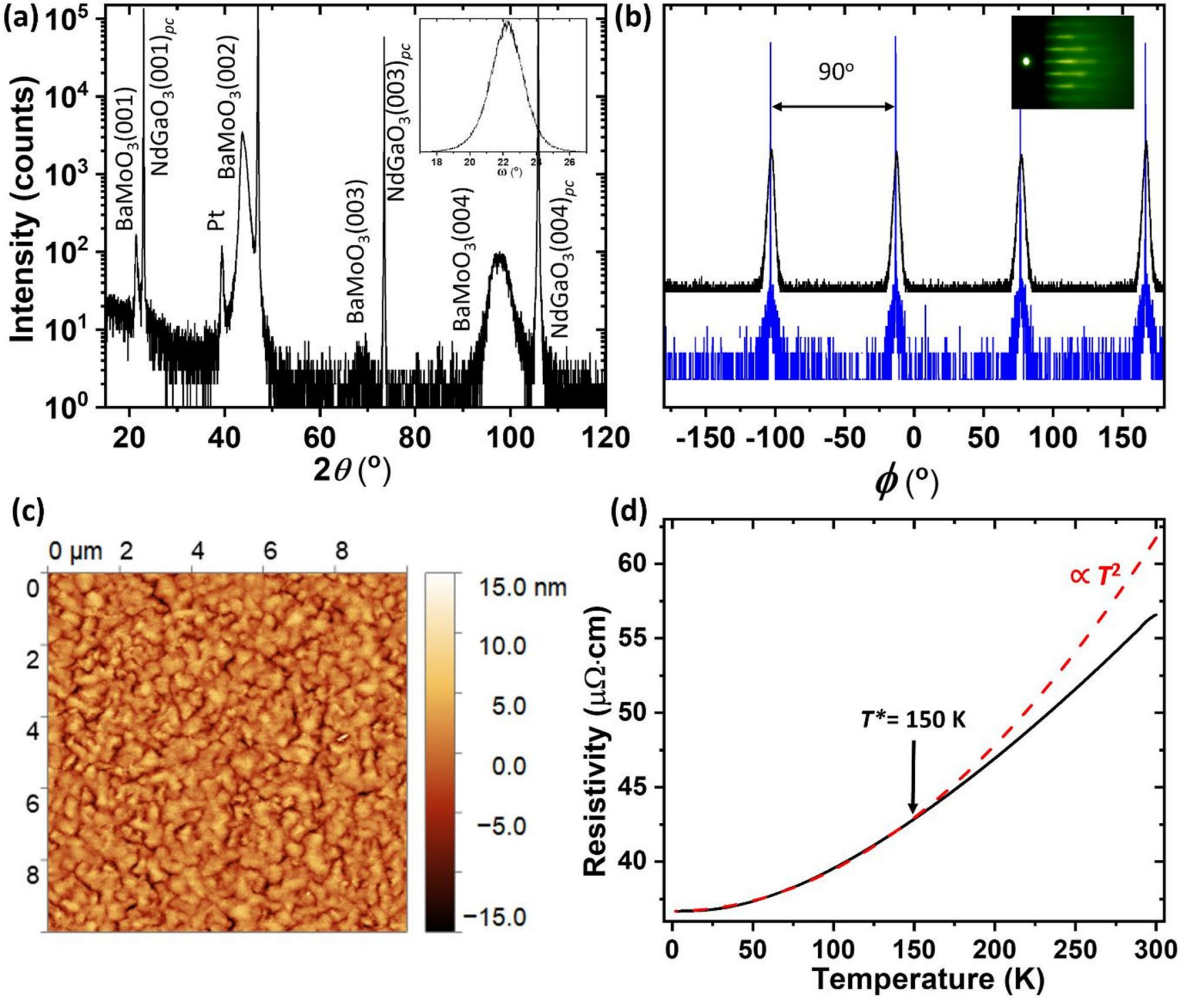
Characterization of BaMoO_3_ film grown on NdGaO_3_(001)_*pc*_ substrate. XRD data in (**a**) symmetric 2*θ*-*ω* scan and (**b**) *f* scans of film and substrate (110) peaks show single phase and single orientation perovskite BaMoO_3_(001) film epitaxially grown on NdGaO_3_(001)_*pc*_ substrate. The Pt peak in (**a**) originates from the sputtered Pt contacts for electrochemical measurements. The RHEED pattern, which was taken along the NdGaO_3_[010]_*pc*_ direction, in the inset in (**b**) shows a streaky pattern, resulting from the highly crystalline film with moderate surface roughness. The inset in (**a**) shows the rocking curve of BaMoO_3_(002). (**c**) AFM image shows the surface morphology of the film with a root mean square roughness of 3 nm. (**d**) Resistivity measurement indicates that the grown BaMoO_3_(001) film is metallic as the resistivity decreases with temperature, and the film has a low resistivity of 56 µΩ·cm at 300 K. The dashed red curve is a fit of the data with T^2^ dependency in the range of 2–300 K.

BaMoO_3_ thin films were grown by PLD on single crystalline NdGaO_3_(001)_*pc*_ substrates. The films were about 700 nm thick according to the thickness analysis in Figure S2a of the SI. They were single phase and oriented in (001) direction with both out-of-plane and in-plane lattice constants of 4.04 Å, relaxing to the bulk value of BaMoO_3_^20^ (Fig. 1a and Figure S2b). The data confirm that no lattice strain is present in the 700 nm BaMoO_3_ film. The relatively large full-width-at half-maximum of the rocking curve of the (002) diffraction of 2.2° (inset Fig. 1a) is likely due to the large lattice mismatch of 4.7% between BaMoO_3_ (*a*_*bulk*_ = 4.04 Å)^20^ and NdGaO_3_(001)_*pc*_ (*a*_pc_ = 3.86 Å)^21^. This growth phenomenon is similar to the one reported of (La_x_Ba_1−x_)SnO_3_ thin films on single crystal SrTiO_3_ substrates with large lattice mismatch, where the film grows in a relaxed manner and the crystallinity is not hampered^22^. In addition, *Φ* scans of (011) diffraction peaks of the film and the substrate (Fig. 1b) show that four peaks, each 90° apart, can be observed at the same *Φ* positions for both film and substrate, confirming the four-fold symmetry of BaMoO_3_ on the NdGaO_3_(001)_*pc*_ substrate.

The inset of Fig. 1b shows a streaky RHEED pattern, which was taken along NdGaO_3_[010]_*pc*_ direction, of the BaMoO_3_(001) film, indicating that the film is crystalline, with moderate surface roughness. This is consistent with the AFM image of the film surface with a root mean square roughness of 3 nm (Fig. 1c).

The electrical measurements show the temperature-dependent resistivity of a typical correlated metal with the critical temperature T* = 150 K (Fig. 1d). Below T*, the electron-electron scattering is dominating the resistivity, as shown by a T^2^ dependence of the resistivity. The BaMoO_3_ film has a resistivity of 56 µΩ·cm at 300 K, which is a very low resistivity compared to other oxide thin films, such as LaNiO_3_ with a resistivity of about 300 µΩ·cm^19^ and comparable to BaMoO_3_ powder (~ 50–100 µ·cm)^20^. Hence, according to XRD, AFM, and resistivity data, it is evident that we have successfully grown single phase, smooth, and metallic BaMoO_3_(001) thin films on single crystalline NdGaO_3_(001)_*pc*_ substrates.

### Degradation of BaMoO_3_ thin films under HER conditions

**Fig. 2:**
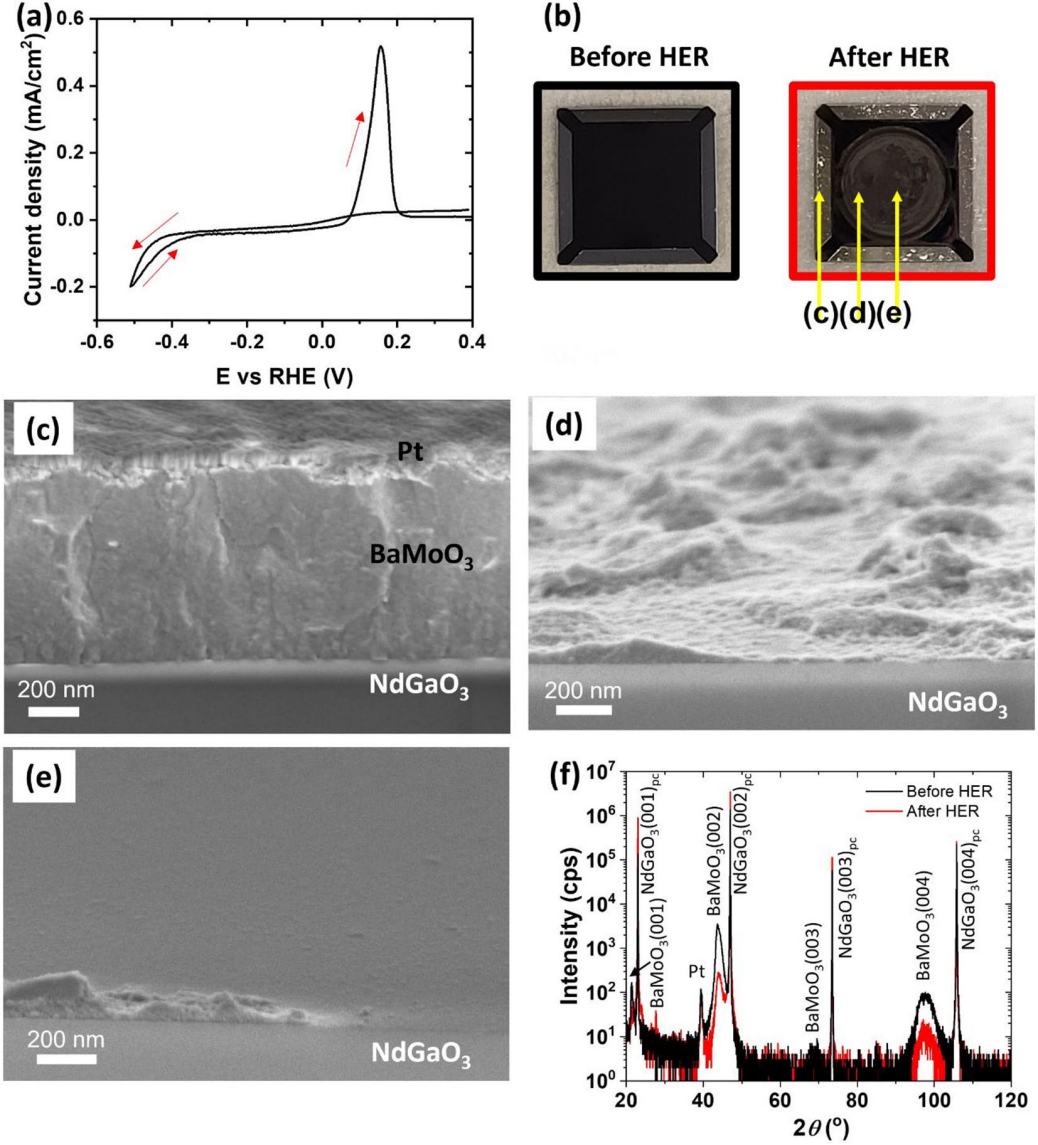
Degradation of single phase BaMoO_3_ thin film on a single crystal NdGaO_3_(001)_*pc*_ substrate under HER conditions. Panel (**a**) shows the CV curve of a 700 nm thick single phase BaMoO_3_(001) thin film measured in 0.1 M KOH. The peak at 0.17 V vs. RHE is probably due to the further oxidation of the remaining BaO_x_-MoO_y_ phases during the anodic scan. The current density is normalized to the exposed geometric area. Panel (**b**) shows an optical image of the 700 nm thick BaMoO_3_ (001) thin film sample before and after 1 CV cycle under HER conditions with locations of high-resolution SEM cross-section images marked. (**c**) SEM in the Pt-contact area (no electrolyte contact). (**d**) SEM in the degraded area near the O-ring edge. (**e**) SEM in the degraded center of the electrolyte-exposed area of the sample. Panel (**f**) shows XRD patterns of the film before and after 1 CV cycle.

The electrocatalytic activity of single phase BaMoO_3_ thin films for HER was assessed using RDE measurements in a three-electrode configuration in 0.1 M KOH electrolyte. CV data of the first measurement cycle shows that the HER current density is as low as -0.05 mA/cm^2^ at -0.4 V vs. RHE (Fig. 2a). This is substantially lower than the current density measured from wet chemically processed BaMoO_3_ which was found to be -15 mA/cm^2^ at -0.4 V vs. RHE^10^. Differences in surface area and exposed crystal facets, as well as the effects of binders and supports may contribute to the differences in HER activity of BaMoO_3_^15^. Such differences in activity between thin film and powder-based films have also often been observed for OER catalysts^16^.

During the CV anodic scan (i.e., the reverse sweep), a clear colour change of the electrolyte-exposed dark-red film is observed when the sample was taken out of the RDE set-up after only 1 CV cycle under HER conditions in KOH electrolyte (Fig. 2b). These results might point to an irreversible reaction of the BaMoO_3_ catalyst. The open-circuit voltage before CV was around 0.4 V vs. RHE, indicating that the film was not changed by going to anodic potential.

The CV-cycled sample was cut cross-sectionally and investigated at different locations (Fig. 2b) using high resolution SEM (Fig. 2c–e). In the Pt-contact area (location c) where no contact with the electrolyte was present, the BaMoO_3_ film appears intact and depicts thickness of 700 nm, shown in Fig. 2c. Near the edge of the O-ring (location d), the BaMoO_3_ film thickness drastically decreased, forming a wave-type-morphology film (Fig. 2d). At the center of the electrolyte-exposed area (location e), the film almost completely disappeared (Fig. 2e). These changes of the BaMoO_3_ film are due to an irreversible reaction and dissolution. The irreversible reaction may be related to the oxidation of Mo^3+^ to Mo^*n*>3+^, leading to the instability of BaMoO_3_ perovskite structure and eventually to the dissolution of BaMoO_3_ into the electrolyte. This phenomenon is somewhat analogous to the irreversible oxidation and dissolution of SrRuO_3_ catalysts where Ru^4+^ is oxidized to Ru^*n*>4+^ at high OER potential^23^. Figure S2c-d, where the BaMoO_3_ film was pulled out at 0 V vs. RHE during the anodic scan, indicates that the irreversible reaction and dissolution of BaMoO_3_ film occur already under HER conditions (below 0 V vs. RHE) and are not directly connected to the peak at 0.17 V vs. RHE in the anodic scan. We suspect that the peak is probably due to the further oxidation of the remaining BaO_x_-MoO_y_ phases (see Figure S3-4) during the anodic scan.

ICP-MS analyses of the electrolyte further support the irreversible reaction and dissolution of BaMoO_3_. The pristine 0.1 M KOH electrolyte has only trace amount of Mo (0.2 µg/L) and Ba (4 µg/L) which originate from impurities in the KOH pellets used for making the electrolyte. The electrolyte after 1 CV cycle of 700 nm BaMoO_3_ film under HER conditions, in contrast, has significant amounts of Mo (447 µg/L) and Ba (117 µg/L). XRD patterns (Fig. 2f) taken before and after HER experiments, show a large decrease in the intensity of BaMoO_3_(001) peaks after HER. The BaMoO_3_(001) peaks are not fully vanished despite apparent loss of BaMoO_3_ in the electrolyte-exposed area due to the large footprint of the X-ray incident beam which measures also the unreacted BaMoO_3_ film outside the electrolyte-exposed area. We suspect that the BaMoO_3_ perovskite phase ceases to exist in the exposed area after 1 CV cycle.

In summary, the fabricated submicron thick BaMoO_3_ films in this study rapidly degraded within only 1 CV cycle under HER conditions due to the irreversible reaction and dissolution of BaMoO_3_ into the electrolyte. It is worth noting that BaMoO_3_ films from micro-sized powder was reported to be stable because the HER activity in KOH electrolyte did not change within 1000 CV cycles (cathodic potential − 0.4 V vs. RHE)^10^. However, this prior work does not report whether the composition of the catalyst or the morphology of the electrode changed. The instability of BaMoO_3_ films in our work is not due to detachment of the BaMoO_3_ thin film from the NdGaO_3_ substrate (Fig. 2), which is often observed in powder-based electrodes. Although there may be differences in defect concentration due to the characteristics of epitaxial thin films and powder-based electrodes, these differences may or may not make a large difference in stability of BaMoO_3_ catalysts. For example, LaNiO_3_ OER catalysts are stable in OER conditions regardless of application in powder or thin film geometry^24^. We also deposited single out-of-plane oriented BaMoO_3_(001) films on native SiO_2_/Si substrates (see Sect. 3 in SI), employing oxide nanosheets as a growth template^25,26^. These films have more extended defects than the single-crystalline films described above due to the grain-boundaries at the nanosheet intersections, offering a point of comparison of BaMoO_3_ films with differing defect properties. The same degradation was found under HER conditions (Figure S3 and Figure S4 in the SI). Such difference in the (in)stability of BaMoO_3_ in our work and the reported study in the literature^10^ may be related to particular instability of the selected (001) facet in our study, the amount of BaMoO_3_ powder catalyst, or the effects of binders and supporting materials. Further studies are needed to shed light on the origins of the contrasting observations. Regarding the intrinsic instability and low activity of the BaMoO_3_(001) facet, it raises the general question whether BaMoO_3_ also as multi-facet powder catalyst can at all serve as a potential and stable catalyst for HER in KOH electrolyte. Future study on the other facets of BaMoO_3_ catalyst will give more insights about the HER performance of BaMoO_3_ catalyst.

## Conclusions

Single phase, smooth, and metallic BaMoO_3_ (001) thin films were successfully grown on single crystalline NdGaO_3_(001)_*pc*_ substrates by pulsed laser deposition. Significant changes in phase and film thickness revealed rapid degradation of the BaMoO_3_ thin films under HER conditions in KOH electrolyte. This was not expected from earlier studies on BaMoO_3_ electrodes fabricated by wet chemical processing from powders^10^. Crystal orientation and / or additives used in the processing, such as binders, might be responsible for the different findings. Further studies are needed to clarify this.

This study demonstrates that thin film systems are a useful tool to uncover intrinsic properties of catalysts for HER. This work also raises caution for drawing conclusions about the properties of catalysts from the electrochemical performance alone. Characterization of catalysts and derivation of intrinsic activity and stability metrics should not be based on electrochemical measurements alone, but need to be complemented with further materials characterization techniques, such as SEM, XRD, and ICP-MS^27^, including characterization after aging and degradation protocols. In addition, this study confirms that thin films and powder based films of the same composition often differ in orientation, crystallinity, additives, such as binders, temperature treatment. This might have tremendous effect not only on the activity, but also on the stability and might explain different performance for same material compositions. Stability is often reported as decay in performance, but the reasons and the mechanisms of degradation need to be studied in more detail to reveal e.g. preferential dissolution of individual facets. Such insides might help designing more stable electrodes. For this, thin films and wet chemical processed films need to be investigated in conjunction in order to make high performing electrodes.

## Supplementary Information

Below is the link to the electronic supplementary material.

Supplementary Material 1

## Data Availability

The data that support the findings of this study will be available upon reasonable request from corresponding authors, P.T.P.L and C.B.
