# Supplementary material for: In search for non precious metal oxide electrodes with the case of BaMoO3 thin films for hydrogen evolution reaction
Source: Sci Rep. 2025 Oct 29;15:37801. doi: 10.1038/s41598-025-21707-x (PMC12572187; doi:10.1038/s41598-025-21707-x)
Supplement: Supplementary file 1 — Supplementary Material 1 [file 41598_2025_21707_MOESM1_ESM.docx]

**Supporting Information**

**In search for non precious metal oxide electrodes with the case of BaMoO_3_ thin films for the hydrogen evolution reaction**

Phu Tran Phong Le^1,2*^, Vadim Ratovskii^1^, Anja Bieberle-Hütter^2^, Gertjan Koster^1^, Christoph Baeumer^1,3*^.

^1^ MESA+ Institute for Nanotechnology, University of Twente, PO Box 217, 7522 NH Enschede, The Netherlands

^2^ Dutch Institute for Fundamental Energy Research (DIFFER), P.O. Box 6336, 5600 HH Eindhoven The Netherlands

^3^ Peter Gruenberg Institute 7, Forschungszentrum Juelich GmbH, 52425 Juelich, Germany and JARA-FIT, RWTH Aachen University, 52056 Aachen, Germany

*Corresponding author emails: [t.p.p.le@utwente.nl](mailto:t.p.p.le@utwente.nl)

[c.baeumer@utwente.nl](mailto:c.baeumer@utwente.nl)

Keywords: catalysts, electrolysis, perovskite oxides, hydrogen evolution reaction, degradation, thin films

1. **Sample configuration and electrical connections for rotating disk electrode (RDE) measurements**

Figure S1 shows the sample configuration and electrical connections ^1^ for HER measurements in rotating disk electrode (RDE) measurements.


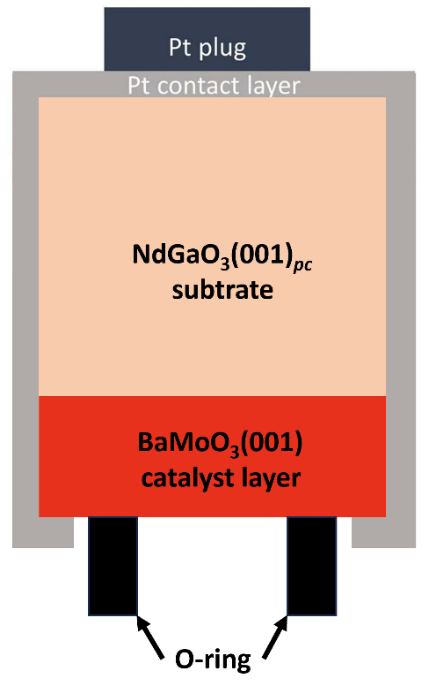


Figure S1. The schematic of sample configuration and electrical connections for HER in RDE [1].

1. **Thickness analysis, reciprocal space mapping of BaMoO_3_ thin film and a control experiment of BaMoO_3_ during HER**

The thickness of the as-deposited BaMoO_3_ film was determined using a cross-section SEM image. The image was taken under the Pt contact at a location where the BaMoO_3_ film was not exposed to the electrolyte during HER (Figure S2a). The film thickness at this location is well representative for the as-deposited BaMoO_3_ thin film.

The reciprocal space mapping around (103) reflections of NdGaO_3_(001)*_pc_* substrate and 700 nm BaMoO_3_(001) film is shown in Figure S2b. It indicates the film is not strained on the substrate and is fully relaxed to the BaMoO_3_ bulk value of 4.04 Å for both out-of-plane and in-plane lattice constants.

Another 700 nm BaMoO_3_ film on NdGaO_3_(001)_pc_ substrate was cycled under HER conditions, and it was pulled out at 0 V vs RHE during anodic scan. A clear colour change of the electrolyte-exposed dark-red film is also observed. XRD patterns of this BaMoO_3_ sample before and after such experiment show similar features as the BaMoO_3_ sample described in the main text.


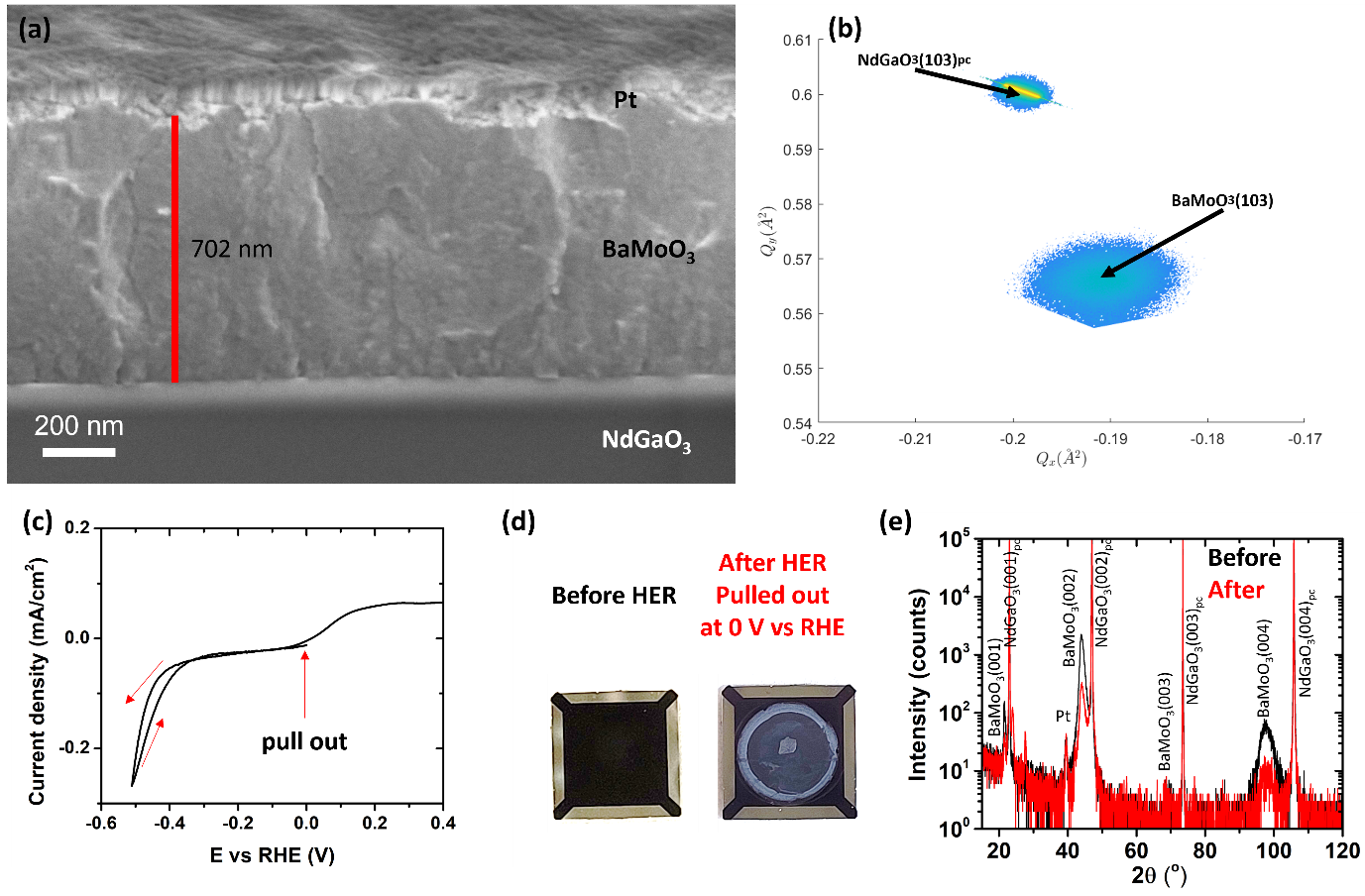


Figure S2. (a) The thickness of the BaMoO_3_ film was determined using a cross-section SEM image. (b) The reciprocal space mapping around (103) reflections of NdGaO_3_(001)*_pc_* substrate and 700 nm BaMoO_3_(001). A control experiment of BaMoO_3_ where the sample was pulled out of the electrolyte at 0 V vs RHE during the anodic scan: (c) CV curve, (d) optical images and (e) XRD patterns of the sample before and after the experiment.

1. **Degradation of BaMoO_3_ films on Ca_2_Nb_3_O_10_ nanosheets on Si**

Oxide nanosheets, which are 2-dimensional micro-sized single crystals with a large library of crystal structures, can serve a similar role as single crystalline substrates for the growth of locally epitaxial oxide thin films with single out–of–plane orientation.^2–5^ Oxide nanosheets are a cheap alternative to avoid the usage of expensive single crystalline substrates. They can be deposited on large surfaces, for example 8-inch Si wafers, as well as on amorphous transparent substrates, like glass, using Langmuir-Blodgett method.^6^ In this work, Ca_2_Nb_3_O_10_ nanosheets were used for the growth of single out-of-plane oriented BaMoO_3_(001) films, which are considered to have more defects compared to BaMoO_3_(001) films on single crystalline NdGaO_3_(001)_pc_ substrates due to the grain-boundary of oxide nanosheets. The detailed preparation and deposition of Ca_2_Nb_3_O_10_ nanosheets on Si were described elsewhere.^7^ The growth conditions of BaMoO_3_ on these nanosheets were the same as on single crystalline NdGaO_3_(001)_pc_ substrates as described in the manuscript. Figure S3 shows the results from materials characterizations of BaMoO_3_ before and after HER.


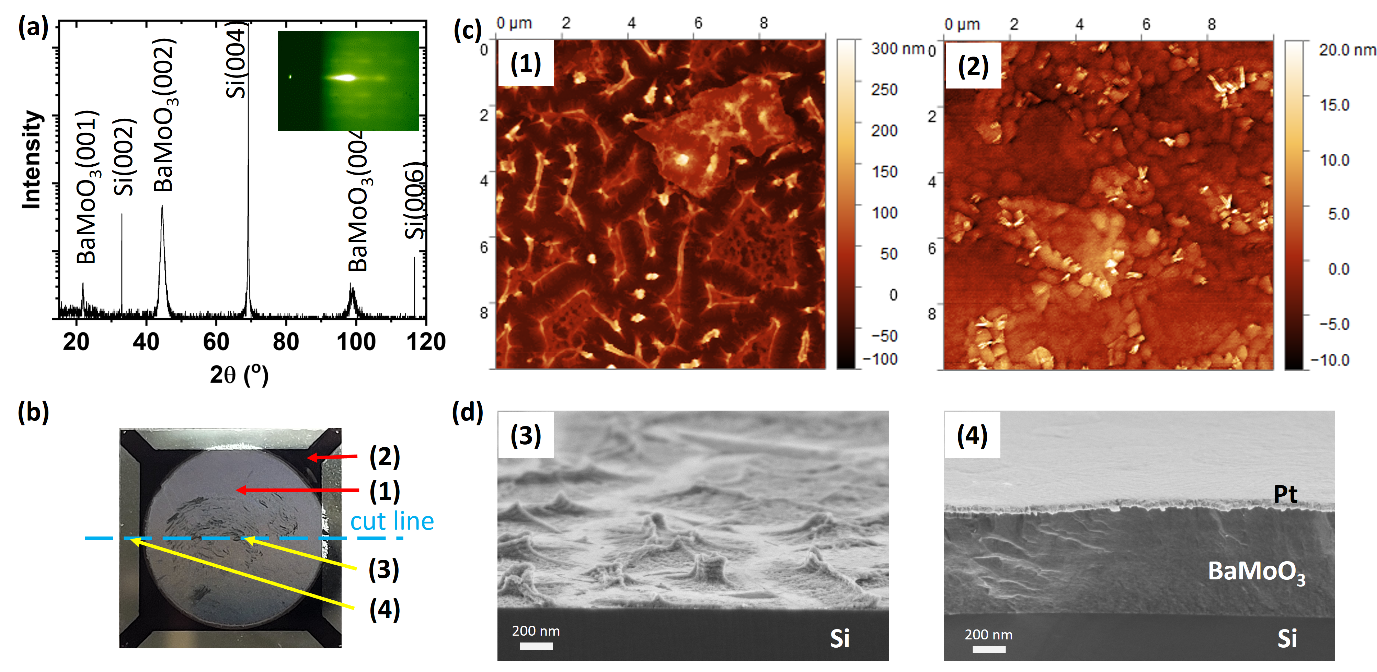


Figure S3. Degradation of 700 nm BaMoO_3_ thin film on Ca_2_Nb_3_O_10_ nanosheets on Si under HER conditions. (a) XRD shows single out-of-plane oriented perovskite BaMoO_3_(001) due to the induced directional growth of Ca_2_Nb_3_O_10_ nanosheets. The RHEED pattern in the inset shows a streaky pattern, resulting from the highly crystalline thin film. (b) Optical image of a 700 nm thick BaMoO_3_(001) thin film sample after one CV cycle under HER conditions. The grey circular area was exposed to HER; numbers refer to the locations where AFM and cross-sectioned SEM were performed; the cut line indicates where the sample was cut for cross-sectional SEM imaging. (c) AFM and (d) high-resolution cross-sectional SEM images at different locations indicate the rapid degradation of BaMoO_3_ forming a wave-like morphology of different stoichiometry. (1) AFM in the degraded center. (2) AFM in the pristine area (no electrolyte contact). (3) cross-sectional SEM image in degraded center. (4) cross-sectional SEM image in the Pt-contact area (no electrolyte contact).

The EDX results (Figure S4) show that the remaining film in the exposed BaMoO_3_ film area (location 3, Figure S3b) had a composition of Ba:Mo:O ratio of 1:1.8:11.3, whereas the unreacted BaMoO_3_ area (location 4, Figure S3b) had a composition of 1:0.9:2.9.


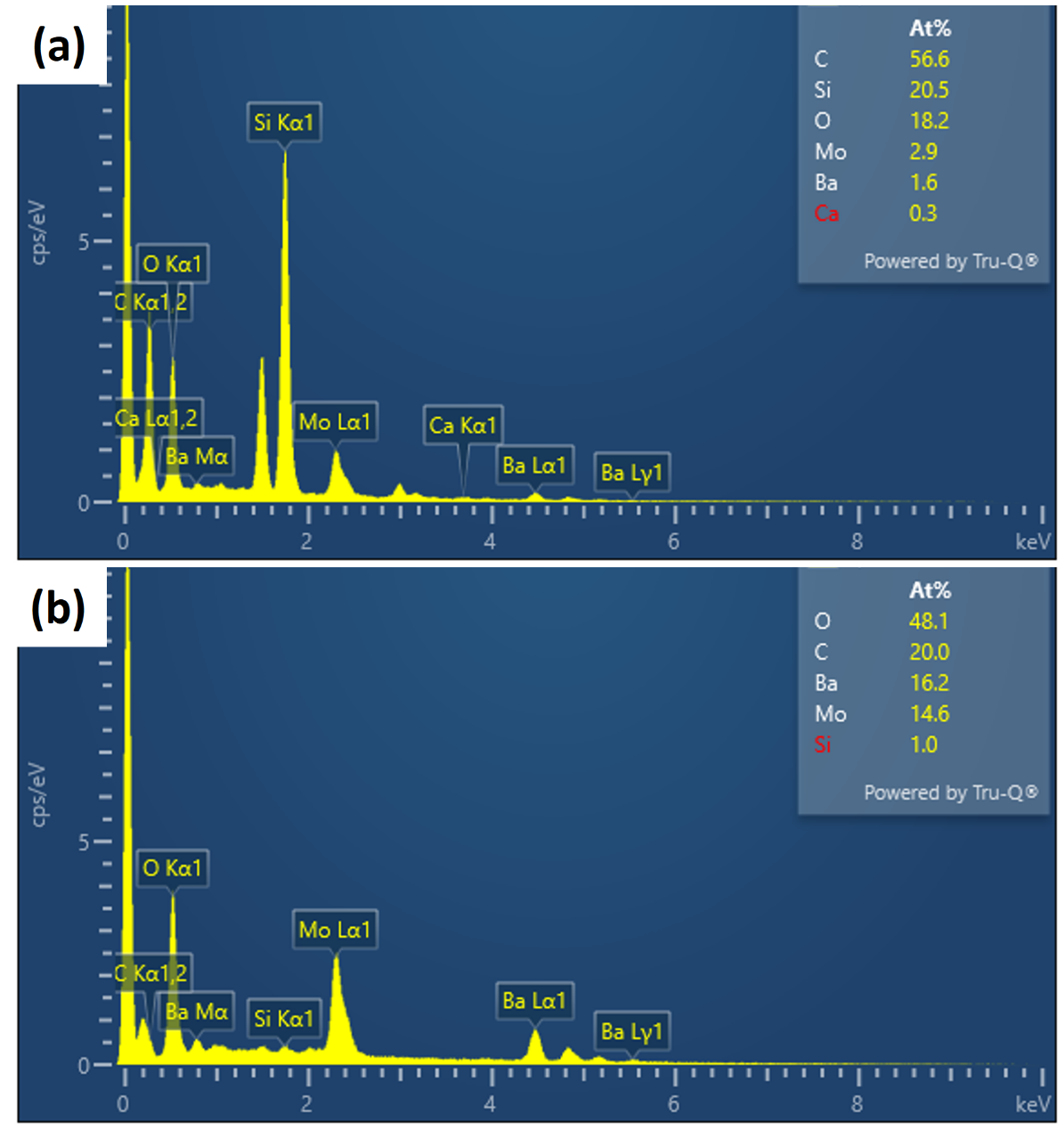


Figure S4. Comparison of the composition of 700 nm BaMoO_3_ film before and after CV in HER conditions: (a) and (b) show EDX data of BaMoO_3_ film in the exposed area (location 3, Figure 3b) and unreacted area (location 4, Figure 3b), respectively.

**References**

1. Baeumer, C. *et al.* Tuning electrochemically driven surface transformation in atomically flat LaNiO3 thin films for enhanced water electrolysis. *Nat. Mater.* **20**, 674–682 (2021).

2. Shibata, T. *et al.* Versatile van der Waals epitaxy-like growth of crystal films using two-dimensional nanosheets as a seed layer: orientation tuning of SrTiO3 films along three important axes on glass substrates. *J. Mater. Chem. C* **2**, 441–449 (2013).

3. Nguyen, M. D. *et al.* Highly Oriented Growth of Piezoelectric Thin Films on Silicon Using Two-Dimensional Nanosheets as Growth Template Layer. *ACS Appl. Mater. Interfaces* **8**, 31120–31127 (2016).

4. Le, P. T. P. *et al.* Tailoring Vanadium Dioxide Film Orientation Using Nanosheets: a Combined Microscopy, Diffraction, Transport, and Soft X-Ray in Transmission Study. *Advanced Functional Materials* **30**, 1900028 (2020).

5. Le, P. T. P., Huang, S., Nguyen, M. D., ten Elshof, J. E. & Koster, G. Tuning the metal insulator transition of vanadium dioxide on oxide nanosheets. *Applied Physics Letters* **119**, 081601 (2021).

6. Le, P. T. P. *et al.* Correlated Metals Transparent Conductors with High UV to Visible Transparency on Amorphous Substrates. *Advanced Materials Interfaces* **10**, 2201335 (2023).

7. Le, P. T. P., ten Elshof, J. E. & Koster, G. Shape Control of Ca2Nb3O10 Nanosheets: Paving the Way for Monolithic Integration of Functional Oxides with CMOS. *ACS Appl. Nano Mater.* **3**, 9487–9493 (2020).
